# Chemokines and immunity

**DOI:** 10.1590/S1679-45082015RB3438

**Published:** 2015

**Authors:** Diana Carolina Torres Palomino, Luciana Cavalheiro Marti

**Affiliations:** 1 Hospital Israelita Albert Einstein, São Paulo, SP, Brazil.; 2 Allergy and Immunopathology Graduate Program of Faculdade de Medicina, Universidade de São Paulo, São Paulo, SP, Brazil.

**Keywords:** Chemokines/immunology, Cytokines/immunology, Immunity

## Abstract

Chemokines are a large family of small cytokines and generally have low molecular weight ranging from 7 to 15kDa. Chemokines and their receptors are able to control the migration and residence of all immune cells. Some chemokines are considered pro-inflammatory, and their release can be induced during an immune response at a site of infection, while others are considered homeostatic and are involved in controlling of cells migration during tissue development or maintenance. The physiologic importance of this family of mediators is resulting from their specificity − members of the chemokine family induce recruitment of well-defined leukocyte subsets. There are two major chemokine sub-families based upon cysteine residues position: CXC and CC. As a general rule, members of the CXC chemokines are chemotactic for neutrophils, and CC chemokines are chemotactic for monocytes and sub-set of lymphocytes, although there are some exceptions. This review discusses the potential role of chemokines in inflammation focusing on the two best-characterized chemokines: monocyte chemoattractant protein-1, a CC chemokine, and interleukin-8, a member of the CXC chemokine sub-family.

## INTRODUCTION

Chemokines constitute a large family of small cytokines and generally have low molecular weight ranging from 7 to 15kDa. Chemokines and their receptors are able to control the migration and residence of all immune cells. Some chemokines are considered pro-inflammatory and their release can be induced during an immune response at a site of infection, while others are considered homeostatic, and are involved in the control of cells migration during tissue development or maintenance. The physiologic importance of this family of mediators is resulting from their specificity; members of the chemokine family induce recruitment of well-defined leukocyte subsets.^1,2^

Many chemokines were initially identified by subtraction hybridization as an immediate or early response genes induced by growth factors. Based on this property, it was supposed that chemokines were involved in cellular proliferation and acted as nuclear factors. However, when complete amino acid sequences were deduced, it was clear that chemokines were secretory proteins.

There are two families of chemokines based on the first cysteine residue: the family called CC chemokines (Chart 1), also known as beta-chemokines. The genes encoding the CC chemokines are located on chromosome 17. CC chemokines stimulate mainly monocytes, but also basophils, eosinophils, T-lymphocytes, and natural killer (NK) cells. The other family CXC (Chart 2), known as alpha-chemokines, have an intervening amino acid between the first two cysteines and are located in the chromosome 4.^3-5^These chemokines, which mainly stimulate neutrophil chemotaxis, contain a Glu-Leu-Arg (ELR) sequence in the N-terminus that is essential for receptor binding.^6,7^

**Chart 1:** Chemokines: CC, receptors and immune function

Chemokine	Other names	Receptor	Immune function
CCL1	I-309	CCR8	Th2 and Treg trafficking
CCL2	MCP-1	CCR2	Monocyte trafficking
CCL3	MIP-1α	CCR1, CCR5	Macrophage-NK migration; T cell/DC interaction
CCL4	MIP-1β	CCR5	Macrophage-NK migration; T cell/DC interaction
CCL5	RANTES	CCR1, CCR3, CCR5	Macrophage-NK migration; T cell/DC interaction
CCL6	C-10	Unknown	?
CCL7	MCP-3	CCR2, CCR3	Monocyte mobilization
CCL8	MCP-2	CCR1, CCR2, CCR3, CCR5	Th2 response
CCL9	MIP-1γ	Unknown	?
CCL10	MIP-1γ	Unknown	?
CCL11	Eotaxin	CCR3	Eosinophil and basophil migration
CCL12	MCP-5	CCR2	Monocyte trafficking
CCL13	MCP-4	CCR2, CCR3, CCR5	Th2 response
CCL14	HCC-1	CCR1	?
CCL15	HCC-2	CCR1, CCR3	?
CCL16	HCC-4	CCR1, CCR2, CCR5	?
CCL17	TARC	CCR4	Th2 response, Th2 cell migration, Treg, lung and skin homing
CCL18	PARC	CCR8	Th2 response, marker AAM, skin homing
CCL19	MIP-3β	CCR7	T cell and DC homing to lymph node
CCL20	MIP-3α	CCR6	Th17 response, B cell and DC homing to gut associated lymphoid tissue
CCL21	SLC	CCR6, CCR7	T cell and DC homing to lymph node
CCL22	MDC	CCR4	Th2 response, Th2 cell migration, T reg migration
CCL23	MIP-3	Unknown	?
CCL24	Eotaxin-2	CCR3	Eosinophil and basophil migration
CCL25	TECK	CCR9	T cell homing to gut, thymocyte migration
CCL26	Eotaxin-3	CCR3, CX3CR1	Eosinophil and basophil migration
CCL27	CTAK	CCR10	T cell homing to skin
CLL28	MEC	CCR3, CCR10	T cell and IgA plasma cell homing to mucosa

Source: Murphy PM et al.^(3)^

**Chart 2:** Chemokines: CXC, receptors and immune function

Chemokine	Other names	Receptor	Immune function
CXCL1	GROα	CXCR2	Neutrophil trafficking
CXCL2	GROβ	CXCR2	Neutrophil trafficking
CXCL3	GROγ	CXCR2	Neutrophil trafficking
CXCL4	PF4	?	Pro-coagulant
CXCL5	ENA78	CXCR2	Neutrophil trafficking
CXCL6	GCP-2	CXCR1, CXCR2	Neutrophil trafficking
CXCL7	NAP-2	CXCR2	Neutrophil trafficking
CXCL8	IL-8	CXCR1, CXCR2	Neutrophil trafficking
CXCL9	MIG	CXCR3	Th1 response, Th1, CD8 and NK trafficking
CXCL10	IP-10	CXCR3	Th1 response, Th1, CD8 and NK trafficking
CXCL11	I-TAC	CXCR3	Th1 response, Th1, CD8 and NK trafficking
CXCL12	SDF-1	CXCR4	Bone marrow homing
CXCL13	BLC	CXCR5	B cell and Tfh positioning in lymph node
CXCL14	BRAK	?	Macrophage skin homing
CXCL15	Lungkine	?	?
CXCL16	5R-PSOX	CXCR6	NKT and ILC migration and survival
CXCL17	DMC	?	?
XCL1	SCM-1α	XCR1	Cross-presentation by CD8+ DC
XCL2	SCM-1β	XCR2	Cross-presentation by CD8+ DC
CX3CL1	Fractalkine	CX3CR1	NK, monocyte and T cell migration

Source: Murphy PM et al.^(3)^

Chemokine receptors are differentially expressed by all leukocytes and can be divided into two groups: G protein-coupled chemokine receptors, which is activated by pertussis toxin (PTX)-sentive G_i_-type G protein; and an atypical chemokine receptors, which appear to form chemokine gradients and dampen inflammation by scavenging chemokines in a G protein independent manner.^8^

Nowadays approximately 50 endogenous chemokines ligands and 20 G-protein-coupled receptors have been described. However, there is still a lot to be comprehended in this field, particularly regarding mechanisms during diseases development. In this review, we focused on a timeline basic knowledge in chemokines.

### MONOCYTE CHEMOATTRACTANT PROTEIN-1 (CCL2/MCP-1) AND CC CHEMOKINES

Acute inflammation is a complex process characterized by the coordinated migration of effector cells to the site of inflammation, as well as displacement of immune cells from peripheral sites to draining lymphoid organs to initiate immune response. Such coordinated movement of cells requires the induced expression of inflammatory chemokines and their respective receptors on target cells. The interaction between chemoattractant and immune cells trigger a series of biochemical and cellular coordinate events.^8,9^

CCL2 is typically secreted in two predominant forms with similar molecular weights of 9 and 13kDa. They have the same protein core and differ by the addition of O-linked carbohydrates to the larger form.^10^ Despite this difference, they have identical activities *in vitro.* ^11^

CCL2 stimulates chemotaxis of monocytes and several cellular events associated with chemotaxis, including Ca^2^ flux and integrin expression. It is also a weak inducer of cytokine expression in monocytes and, at high concentrations, elicits a respiratory burst leading to generation of reactive oxygen species.^11,12^

Monocytes utilize different chemoattractant molecules to migrate; however, CCL2 and CCL7 are rapidly produced by stromal cells and immune cells after pattern-recognition receptors (PRR) activation or after cytokine stimulation. CCL2 dimerizes and binds to extracellular matrix glycosaminoglycans, thus establishing a stable gradient for CCR2+ inflammatory monocytes.^8,13^

CCL2 expression has been identified upregulated in certain cell types of some diseases, such as macrophages/foam cells and smooth muscle cells in atherosclerosis; delayed-type of hypersensitivity reactions in the skin mononuclear phagocytes, endothelial cells, and keratinocytes; melanoma cells in skin cancer and synoviocites of rheumatoid arthritis.^14-17^

Two other chemokines structurally related to CCL2 are named CCL8 (MCP-2) and CCL7 (MCP-3). Generally, CCL2 and CCL7 have similar properties, although CCL7 may exhibit characteristics from both CCL2 and CCL5 (RANTES), suggesting that it has a broader range of chemokine binding. Others CC chemokines include macrophage inflammatory protein 1α (MlP-1α/CCL3), macrophage inflammatory protein-1β (MIP-1β/CCL4), T-cell activation gene-3 (TCA-3/CCL1), and CCL5, which support and regulate activated T-cells.^12,18,19^

Eosinophil is a leukocyte present in high numbers during inflammatory allergic reactions, such as asthma and rhinitis; there is evidence that links accumulation and activation of these cells with tissue injury and lung dysfunction.^20-22^ Humbles et al. purified and sequenced a CC-chemokine from bronchoalveolar lavage fluid of allergen-challenged sensitized animal model. They also identified CCL11 (Eotaxin-1) as a local chemoattractant responsible for eosinophil recruitment, via CCR3, a chemokine receptor highly expressed on eosinophils.^23^ These leukocytes express CCR1 and CCR3 and can respond to a variety of chemokines including CCL11, CCL24 (Eotaxin-2) and CCL26 (Eotaxin-3).^24,25^

CCL19 (MIP-3β) and CCL21 (SLC) are expressed mainly by stromal cells in T cell-rich zones of lymph nodes. They are mediators of homeostatic trafficking of naïve T cells, but also play a role on T cell priming and activation, as well as on lymphocytes recruitment to inflamed tissue. The pathway signaling of both proteins take place via CCR7, which is expressed on T cells and mature dendritic cells. Although CCL19 and CCL21 exhibit the same affinity for CCR7, only CCL19 has ability to desensitize and internalize the receptor.^8^

T cells and progenitor cells enter the thymus in response to CCL21/SLC and CCL25/TECK, which are produced by thymic epithelium and surrounding structures. CCR9 guides these cells to the subcapsular zone. Subsequent thymic maturation from double-positive through single-positive thymocytes occurs in concert with their migration into the medulla, which is dependent on CCR7 and perhaps another chemokine yet to be defined.^26^

In general, chemokines CCL2, CCL3, CCL4, CCL5, CCL11 and CCL13 are classified as inflammatory; CCL18, CCL19, CCL21, CCL25 and CCL27 as homeostatic. CCL1, CCL17, CCL20 and CCL22 with dual function inflammatory and homeostatic while CCL14, CCL15, CCL16 and CCL23 are related with plasma or platelet.^27^

### INTERLEUKIN-8 AND CXC CHEMOKINES

lnterleukin-8 (IL-8/CXCL8) is a prototypical member of CXC chemokine family. The IL-8 gene encodes a 99-aminoacid translation product. It undergoes proteolytic cleavage of the amino terminal yielding several different products. IL-8 in solution is thought to exist as non-covalently linked dimer, and, *in vitro*, it is a potent chemoattractant for neutrophils. It has been reported to be also a chemoattractant for a subset of T-lymphocytes.^28^

IL-8 has also been named neutrophil activating protein-1 (NAP-1), since it stimulates release of neutrophil granules. Like many other chemoattractants, IL-8 induces re-arrangement of cytoskeleton, changes in intracellular Ca^2^ levels, activation of integrins, exocytosis of granule proteins, and respiratory burst.^29^There are some chronic inflammatory diseases, such as psoriasis, rheumatoid arthritis, as well as pulmonary diseases, in which IL-8 is overexpressed, and high levels of IL-8 are also seen after septic shock or systemic administration of endotoxin.^30-32^

Regarding other CXC-chemokines members, neutrophils are the main responsive cell type, since they express receptors CXCR1 and CXCR2, which are able to bind to CXCL1, CXCL2, CXCL5, CXCL6, CXCL7 and CXCL8. In acute inflammation initiated by bacterial invasion, neutrophils are the first cells that infiltrate into the tissue. Neutrophils play a critical role during infection control, first by their capacity of microorganism phagocytosis, and second by releasing other chemotactic mediators, such as CXCL1, CXCL8, CXCL9 and CXCL10 that recruit other leucocytes to the affected tissues.^33^

Chemokines control innate immune cell trafficking between the bone marrow, blood, and peripheral tissues during inflammation. At the same time, systemic levels of G-CSF lead to a reduction on CXCL12 production by bone marrow and decreased CXCR4 expression by neutrophils. Release of neutrophil from bone marrow into the blood is triggered by the switch of activated receptor, from CXCR4 to CXCR2.^8^

Mast cells also play an important role in acute inflammation because they express a wide variety of PRR and contain large granules of preformed inflammatory mediators. LPS stimulated murine peritoneal mast cells leads to immediate release of CXCL1- and CXCL2-containing granules, but not histamine-containing granules, as well as transcriptional activation of CXCL1 and CXCL2. This promotes early neutrophil recruitment that is abolished in mast cell-depleted mice, but not macrophage-depleted mice.^8^

CX3CL1 (or fractalkine) is an atypical chemokine with a documented role in the development of numerous inflammatory diseases, including atherosclerosis. It represents the only known member of the CX3C family and was first characterized by Bazan et al.^34^ It is a unique chemotactic factor existing in both membrane-bound and soluble form. The human CX3CL1 molecule consists of 373 amino acids and it is functionally divided into four domains: an extracellular domain of 76 amino acids connected to an extended 241 amino acid mucin-like stalk, followed by transmembrane and intracellular domains of 21 and 35 amino acids, respectively. CX3CL1 is synthesized as a membrane-bound molecule with the chemokine domain presented on a mucin-like stalk, which mediates the direct capture of circulating leukocytes. Cleavage at the base of this stalk by a protease, namely the tumor necrosis alpha-converting enzyme, generates a soluble chemokine, which function as a potent chemoattractant of target cells.^34,35^

The fractalkine receptor CX3CR1 is a well-established marker of anti-inflammatory or patrolling monocytes known by providing pro-survival signal to anti-inflammatory monocytes, but is also present in NK cells, T cells, and smooth muscle cells, where it mediates migration, adhesion and proliferation. CXCR3 is also present on pro-inflammatory monocytes and may play a role on normal function of inflammatory monocytes.^8^

In general, chemokines CXCL1, CXCL2, CXCL3, CXCL5, CXCL6, CXCL7 and CXCL8 are classified as inflammatory; CXCL12 and CXCL13 as homeostatic. CXCL9, CXCL10, CXCL11 and CXCL16 with dual function inflammatory and homeostatic, while CXCL4 is plasmatic or platelet related.^27^
