# Supplementary material for: Chemokines and immunity
Source: Einstein (Sao Paulo). 2015 Jul-Sep;13(3):469–73. doi: 10.1590/S1679-45082015RB3438 (PMC4943798; doi:10.1590/S1679-45082015RB3438)
Supplement: Translation

# Translation: Quimiocinas e imunidade

**Authors:** Diana Carolina Torres Palomino, Luciana Cavalheiro Marti

**Affiliations:** 1 Hospital Israelita Albert Einstein, São Paulo, SP, Brasil.; 2 Programa de Pós-Graduação em Alergia e Imunopatologia da Faculdade de Medicina, Universidade de São Paulo, São Paulo, SP, Brasil.

**Keywords:** Quimiocinas/imunologia, Citocinas/imunologia, Imunidade

## Abstract

Quimiocinas são uma grande família de pequenas citocinas e seu peso molecular varia de 7 a 15kDa. As quimiocinas e seus receptores são capazes de controlar a migração e a residência de células imunes. Algumas quimiocinas são consideradas pró-inflamatórias e podem ser induzidas durante a resposta imune no sítio de infecção, enquanto outras são consideradas homeostáticas e estão envolvidas no controle da migração celular durante o desenvolvimento ou a manutenção dos tecidos. A importância fisiológica dessa família de mediadores é resultado de sua especificidade − os membros da família de quimiocinas induzem ao recrutamento de subtipos bem definidos de leucócitos. Existem duas grandes subfamílias de quimiocinas baseadas na posição dos resíduos de cisteínas: CXC e CC. Como regra geral, membros da família de quimiocinas CXC são quimiotáticos de neutrófilos, e as quimiocinas CC são quimiotáticos de monócitos e subtipos de linfócitos, apesar de existirem algumas exceções. Esta revisão discute o potencial papel das quimiocinas na inflamação focando nas duas quimiocinas mais bem caracterizadas: a proteína quimioatraente de monócitos-1, uma quimiocina CC, e a interleucina 8, uma quimiocina membro da subfamília CXC.

## INTRODUÇÃO

As quimiocinas formam uma grande família de pequenas citocinas, geralmente de baixo peso molecular, que varia entre 7 e 15kDa. As quimiocinas e seus receptores são capazes de controlar a migração e a residência de todas as células imunes. Algumas quimiocinas são consideradas pró-inflamatórias e sua liberação pode ser induzida durante uma resposta imune em um sítio de infecção, enquanto outras são consideradas homeostáticas e estão envolvidas no controle da migração celular durante o desenvolvimento ou a manutenção dos tecidos. A importância fisiológica dessa família de mediadores é resultado de sua especificidade. Os membros da família das quimiocinas induzem ao recrutamento de subtipos bem definidos de leucócitos.^1,2^

Muitas quimiocinas foram identificadas inicialmente por meio da hibridização subtrativa, como genes de resposta imediata ou precoce induzidos por fatores de crescimento. Com base nessa propriedade, supôs-se que as quimiocinas estivessem envolvidas na proliferação celular e atuassem como fatores nucleares. No entanto, quando sequências completas de aminoácidos foram deduzidas, ficou claro que as quimiocinas eram proteínas secretoras.

Há duas famílias de citocinas com base no primeiro resíduo de cisteína; a primeira é a família chamada quimiocinas CC (Quadro 1), também conhecidas como beta-quimiocinas. Os genes que codificam as quimiocinas CC estão localizados no cromossomo 17. As quimiocinas CC estimulam principalmente os monócitos, mas também os basófilos, eosinófilos, linfócitos-T e as células *natural killer* (NK). A outra família é a das quimiocinas CXC (Quadro 2), conhecidas como as alfa-quimiocinas, que têm um aminoácido que intervém entre as duas primeiras cisteínas, localizadas no cromossomo 4.^3-5^ Essas quimiocinas, as quais estimulam principalmente a quimiotaxia de neutrófilos, contêm uma sequência Glu-Leu-Arg (ELR) no N-terminal que é essencial para a ligação de receptores.^6,7^

**Quadro 1:** Quimiocinas: CC, receptores e função imune

Quimiocina	Outros nomes	Receptor	Função imune
CCL1	I-309	CCR8	Tráfego de Th2 e Treg
CCL2	MCP-1	CCR2	Tráfego de monócito
CCL3	MIP-1α	CCR1, CCR5	Migração macrófago NK; interação célula T/DC
CCL4	MIP-1β	CCR5	Migração macrófago NK; interação célula T/DC
CCL5	RANTES	CCR1, CCR3, CCR5	Migração macrófago NK; interação célula T/DC
CCL6	C-10	Desconhecido	?
CCL7	MCP-3	CCR2, CCR3	Mobilização de monócito
CCL8	MCP-2	CCR1, CCR2, CCR3, CCR5	Resposta Th2
CCL9	MIP-1γ	Desconhecido	?
CCL10	MIP-1γ	Desconhecido	?
CCL11	Eotaxina	CCR3	Migração de eosinófilo e basófilo
CCL12	MCP-5	CCR2	Tráfego de monócito
CCL13	MCP-4	CCR2, CCR3, CCR5	Resposta Th2
CCL14	HCC-1	CCR1	?
CCL15	HCC-2	CCR1, CCR3	?
CCL16	HCC-4	CCR1, CCR2, CCR5	?
CCL17	TARC	CCR4	Resposta Th2, migração de célula Th2, Treg e *homing *(endereçamento) para pulmão e pele
CCL18	PARC	CCR8	Resposta Th2, marcador AAM e *homing *(endereçamento) para pele
CCL19	MIP-3β	CCR7	Célula T/DC com *homing *(endereçamento) para linfonodo
CCL20	MIP-3α	CCR6	Resposta Th17, célula B e DC com *homing* (endereçamento) para tecido linfoide associado ao intestino
CCL21	SLC	CCR6, CCR7	Célula T e DC com *homing* (endereçamento) para linfonodo
CCL22	MDC	CCR4	Resposta Th2, migração de célula Th2, migração de T reg
CCL23	MIP-3	Desconhecido	?
CCL24	Eotaxina-2	CCR3	Migração de eosinófilo e basófilo
CCL25	TECK	CCR9	*Homing* (endereçamento) de célula T para intestino, migração de timócito
CCL26	Eotaxina-3	CCR3, CX3CR1	Migração de eosinófilo e basófilo
CCL27	CTAK	CCR10	Célula T com *homing* (endereçamento) para pele
CLL28	MEC	CCR3, CCR10	Célula T e plasmócitos de IgA com *homing* (endereçamento) mucosa

Fonte: Murphy PM et al.^(3)^

**Quadro 2:** Quimiocinas: CXC, receptores e função imune

Quimiocina	Outros nomes	Receptor	Função imune
CXCL1	GROα	CXCR2	Tráfego de neutrófilos
CXCL2	GROβ	CXCR2	Tráfego de neutrófilos
CXCL3	GROγ	CXCR2	Tráfego de neutrófilos
CXCL4	PF4	?	Pró-coagulante
CXCL5	ENA78	CXCR2	Tráfego de neutrófilos
CXCL6	GCP-2	CXCR1, CXCR2	Tráfego de neutrófilos
CXCL7	NAP-2	CXCR2	Tráfego de neutrófilos
CXCL8	IL-8	CXCR1, CXCR2	Tráfego de neutrófilos
CXCL9	MIG	CXCR3	Resposta Th1, tráfego de Th1, CD8 e NK
CXCL10	IP-10	CXCR3	Resposta Th1, tráfego de Th1, CD8 e NK
CXCL11	I-TAC	CXCR3	Resposta Th1, tráfego de Th1, CD8 e NK
CXCL12	SDF-1	CXCR4	*Homing* (endereçamento) para medula óssea
CXCL13	BLC	CXCR5	Posicionamento de célula B e Tfh no linfonodo
CXCL14	BRAK	?	*Homing* (endereçamento) de macrófago para a pele
CXCL15	Lungkine	?	?
CXCL16	5R-PSOX	CXCR6	Migração e sobrevida de NKT e ILC
CXCL17	DMC	?	?
XCL1	SCM-1α	XCR1	Apresentação cruzada por CD8+ DC
XCL2	SCM-1β	XCR2	Apresentação cruzada por CD8+ DC
CX3CL1	Fractalquina	CX3CR1	Migração de NK, monócito e célula T

Fonte: Murphy PM et al.^(3)^

Os receptores de quimiocinas são expressos de forma diferenciada por todos os leucócitos e podem ser divididos em dois grupos: os receptores de quimiocina acoplados à proteína G, que são ativados pela proteína G do tipo G_i _sensível à toxina pertússis (PTX); e os receptores atípicos de quimiocina, que parecem formar gradientes de quimiocina e reduzir a inflamação ao sequestrarem as quimiocinas de uma forma que não depende da proteína G.^8^

Atualmente, cerca de 50 ligantes endógenos das quimiocinas e 20 receptores acoplados à proteína G já foram descritos. No entanto, ainda há muito por compreender nessa área, especialmente com relação a mecanismos que ocorrem durante o desenvolvimento de doenças. Nesta revisão, nós nos concentramos em uma cronologia de conhecimento básico sobre as quimiocinas.

## PROTEÍNA QUIMIOTÁTICA DE MONÓCITOS 1 (CCL2/MCP-1) E QUIMIOCINAS CC

A inflamação aguda é um processo complexo caracterizado pela migração coordenada de células efetoras para o sítio de inflamação, além do deslocamento de células imunes de locais periféricos até os órgãos linfoides drenantes, para iniciar a resposta imunológica. Essa movimentação coordenada de células exige a expressão induzida de quimiocinas inflamatórias e de seus respectivos receptores nas células-alvo. A interação entre os quimiotáticos e as células imunes desencadeia uma série de eventos bioquímicos e celulares coordenados.^8,9^

A CCL2 é secretada tipicamente em duas formas predominantes, com pesos moleculares semelhantes de 9 e 13kDa. Tem o mesmo núcleo proteico e difere pela adição de carboidratos O-ligados à forma maior.^10^ Apesar da diferença, tem atividades idênticas *in vitro.* ^11^

A CCL2 estimula a quimiotaxia de monócitos e vários eventos celulares associados à quimiotaxia, incluindo o fluxo de Ca^2^ e a expressão de integrinas. Ela é também um indutor fraco da expressão de citocina em monócitos e, em altas concentrações, desperta *respiratory burst*, que gera espécies reativas de oxigênio.^11,12^

Os monócitos utilizam diferentes moléculas quimiotáticas para migrar; entretanto, CCL2 e CCL7 são produzidas rapidamente por células estromais e células imunes após a ativação dos receptores de reconhecimento padrão (PRR) ou depois da estimulação de citocinas. A CCL2 forma um dímero e se liga a glicosaminoglicanos da matriz extracelular, estabelecendo, dessa forma, um gradiente estável para os monócitos inflamatórios CCR2+.^8,13^

A expressão de CCL2 foi identificada aumentada em certos tipos de células de algumas doenças, como nos macrófagos/células espumosas e nas células musculares lisas de aterosclerose; nas reações de hipersensibilidade tardia em fagócitos mononucleares, células endoteliais e nos queratinócitos da pele; nas células de melanoma no câncer de pele e nos sinoviócitos em artrite reumatoide.^14-17^

Duas outras quimiocinas estruturalmente relacionadas à CCL2 são chamadas de CCL8 (MCP-2) e CCL7 (MCP-3). Geralmente, CCL2 e CCL7 têm propriedades semelhantes, apesar da última poder exibir características tanto da CCL2 quanto da CCL5 (RANTES), sugerindo que ela possui maior amplitude de ligação com outras quimiocinas. Outras quimiocinas CC incluem a proteína inflamatória de macrófagos-1α (MlP-1α/CCL3), a proteína inflamatória de macrófagos-1β (MIP-1β/CCL4), o gene-3 de ativação de células T (TCA-3/CCL1), e a CCL5, a qual auxilia e regula as células T ativadas.^12,18,19^

O eosinófilo é um leucócito presente em grandes quantidades durante reações inflamatórias alérgicas, como a asma e a rinite; há indícios que ligam o acúmulo e a ativação dessas células à lesão tecidual e à disfunção pulmonar.^20-22^ Humbles et al. purificaram e sequenciaram uma quimiocina-CC do fluido de lavado broncoalveolar de modelo animal sensibilizado e desafiado com alérgeno. Também identificaram a CCL11 (Eotaxina-1) como um quimiotático local responsável pelo recrutamento de eosinófilos, via CCR3, um receptor de quimiocinas altamente expresso em eosinófilos.^23^ Esses leucócitos expressam CCR1 e CCR3, e podem responder a diferentes quimiocinas, incluindo a CCL11, a CCL24 (Eotaxina-2) e a CCL26 (Eotaxina-3).^24,25^

A CCL19 (MIP-3β) e a CCL21 (SLC) são expressas principalmente por células estromais na zona de células T dos linfonodos. São mediadoras do tráfego homeostático de linfócitos T virgens, mas também têm papel no *priming* e na ativação das células T, bem como no recrutamento de linfócitos para tecidos inflamados. A via de sinalização de ambas as proteínas ocorre por meio do CCR7, o qual é expresso em células T e em células dendríticas maduras. Apesar de CCL19 e CCL21 exibirem a mesma afinidade pelo CCR7, somente CCL19 tem a capacidade de dessensibilizar e internalizar o receptor.^8^

As células T e as células progenitoras entram no timo em resposta a CCL21/SLC e a CCL25/TECK, que geralmente são produzidas pelo epitélio tímico e estruturas adjacentes. O CCR9 guia essas células até a zona subcapsular. A maturação tímica subsequente de timócitos duplo-positivos em timócitos simples-positivos ocorre conjuntamente com sua migração para dentro da medula, que é dependente do CCR7 e talvez de outra quimiocina ainda a ser definida.^26^

Geralmente, as quimiocinas CCL2, CCL3, CCL4, CCL5, CCL11 e CCL13 são classificadas como inflamatórias, enquanto as quimiocinas CCL18 CCL19, CCL21, CCL25 e CCL27 são homeostáticas. As quimiocinas CCL1, CCL17, CCL20 e CCL22 têm dupla função inflamatória e homeostática, enquanto as quimiocinas CCL14, CCL15, CCL16 e CCL23 estão relacionadas ao plasma ou às plaquetas.^27^

## INTERLEUCINA-8 E QUIMIOCINAS CXC

A interleucina 8 (IL-8/CXCL8) é um membro exemplar da família das quimiocinas. O gene IL-8 codifica um produto da tradução de 99 aminoácidos. Ele passa por uma clivagem proteolítica do amino-terminal, resultando em vários produtos diferentes. Acredita-se que a IL-8 em solução exista sob a forma de um dímero ligado de maneira não covalente e, *in vitro*, ela é um quimiotático potente para os neutrófilos. Também há relatos de que é quimiotática para um subgrupo de linfócitos-T.^28^

A IL-8 também já foi chamada de proteína ativadora de neutrófilos-1 (NAP-1), já que estimula a liberação de grânulos neutrófilos. Assim como vários outros quimiotáticos, a IL-8 induz à reorganização do citoesqueleto, alterações nos níveis de Ca^2^ intracelular, ativação de integrinas, exocitose de proteínas granulares e *respiratory burst*.^29^ Há algumas doenças inflamatórias crônicas, como a psoríase, artrite reumatoide, bem como doenças pulmonares, em que há a superexpressão da IL-8. Altos níveis de IL-8 também são observados após choque séptico ou administração sistêmica de endotoxinas.^30-32^

Com relação a outros membros das quimiocinas-CXC, os neutrófilos são o principal tipo de célula responsivo, já que eles expressam os receptores CXCR1 e CXCR2, ao quais podem se ligar a CXCL1, CXCL2, CXCL5, CXCL6, CXCL7 e CXCL8. Em uma inflamação aguda iniciada por invasão bacteriana, os neutrófilos são as primeiras células a infiltrarem os tecidos. Os neutrófilos têm um papel essencial durante o controle da infecção, primeiro devido à sua capacidade de fagocitar microrganismos, e segundo por liberarem outros mediadores quimiotáticos, como CXCL1, CXCL8, CXCL9 e CXCL10, os quais recrutam outros leucócitos para os tecidos afetados.^33^

As quimiocinas controlam o tráfego de células imunes inatas entre a medula óssea, o sangue e os tecidos periféricos durante a inflamação. Ao mesmo tempo, níveis sistêmicos de G-CSF levam à redução da produção de CXCL12 pela medula óssea e à diminuição da expressão de CXCR4 pelos neutrófilos. A liberação de neutrófilos da medula óssea para o sangue é desencadeada pela mudança do receptor ativado de CXCR4 para CXCR2.^8^

Os mastócitos também desempenham um papel importante na inflamação aguda, pois expressam uma grande variedade de PRR e contêm grandes grânulos pré-formados de mediadores inflamatórios. Mastócitos peritoneais de murinos estimuladas por LPS levam à liberação imediata de grânulos contendo CXCL1 e CXCL2, mas não de grânulos contendo histamina, bem como à ativação transcricional de CXCL1 e CXCL2. Isso promove um recrutamento precoce de neutrófilos, que é abolido em camundongos depletados de mastócitos, mas não em animais depletados de macrófagos.^8^

A CX3CL1 (ou fractalquina) é uma quimiocina atípica com um papel documentado no desenvolvimento de várias doenças inflamatórias, incluindo a aterosclerose. Ela representa o único membro conhecido da família CX3C e foi caracterizada pela primeira vez por Bazan et al.^34^ É um fator quimiotático único existente tanto na forma solúvel, quanto na forma ligada à membrana. A molécula CX3CL1 humana consiste de 373 aminoácidos e é funcionalmente dividida em quatro domínios: um domínio extracelular com 76 aminoácidos ligados a uma haste estendida semelhante à mucina com 241 aminoácidos, seguido por domínios transmembrana e intracelulares de 21 e 35 aminoácidos, respectivamente. A CX3CL1 é sintetizada como uma molécula ligada à membrana, com o domínio quimiocina apresentado em uma haste semelhante à mucina, o qual media a captura direta de leucócitos circulantes. Uma clivagem na base dessa haste por uma protease, chamada enzima conversora de fator de necrose tumoral-alfa, gera uma quimiocina solúvel que funciona como um potente quimiotático das células-alvo.^34,35^

O receptor CX3CR1 é um marcador bem estabelecido de monócitos anti-inflamatórios ou circulantes conhecidos por fornecerem sinal pró-sobrevivência para monócitos anti-inflamatórios, mas também está presente em células NK, células T e células musculares lisas, onde mediam migração, adesão e proliferação. O CXCR3 também está presente em monócitos pró-inflamatórios e pode atuar na função normal de monócitos inflamatórios.^8^

Geralmente, as quimiocinas CXCL1, CXCL2, CXCL3, CXCL5, CXCL6, CXCL7 e CXCL8 são classificadas como inflamatórias, enquanto a CXCL12 e a CXCL13 são homeostáticas. As quimiocinas CXCL9, CXCL10, CXCL11 e CXCL16 têm dupla função inflamatória e homeostática, enquanto a CXCL4 está relacionada ao plasma ou às plaquetas.^27^
